# MED13L integrates Mediator-regulated epigenetic control into lung cancer radiosensitivity

**DOI:** 10.7150/thno.48247

**Published:** 2020-07-23

**Authors:** Nasha Zhang, Yemei Song, Yeyang Xu, Jiandong Liu, Yue Shen, Liqing Zhou, Jinming Yu, Ming Yang

**Affiliations:** 1 Shandong Provincial Key Laboratory of Radiation Oncology, Cancer Research Center, Shandong Cancer Hospital and Institute, Shandong First Medical University and Shandong Academy of Medical Sciences, Jinan, Shandong Province, 250117, China.; 2 Department of Radiation Oncology, Shandong Cancer Hospital and Institute, Shandong First Medical University and Shandong Academy of Medical Sciences, Jinan, Shandong Province, 250117, China.; 3 Department of Radiation Oncology, Huaian No. 2 Hospital, Huaian, Jiangsu Province, 223002, China.

**Keywords:** lung cancer radiotherapy, MED13L, miR-4497, Mediator, H3K27ac, PRKCA

## Abstract

To date, efforts to improve non-small-cell lung cancer (NSCLC) outcomes with increased radiation dose have not been successful. Identification of novel druggable targets that are capable to modulate NSCLC radiosensitivity may provide a way forward. Mediator complex is implicated in gene expression control, but it remains unclear how Mediator dysfunction is involved in cancer radiotherapy.

**Methods:** The biologic functions of miR-4497, MED13L and PRKCA in NSCLC radiosensitivity were examined through biochemical assays including gene expression profilling, cell proliferation assay, colony formation assay, wound healing assay, transwell assay, dual luciferase reporter assay, xenograft models, immunoprecipitation, and chromatin immunoprecipitation sequencing. Clinical implications of miR-4497, MED13L and PRKCA in radiosensitivity were evaluated in NSCLC patients treated with concurrent chemoradiotherapy or radiotherapy alone.

**Results:** We found that radiation can trigger disassemble of Mediator complex via silencing of MED13L by miR-4497 in NSCLC. Although not interrupting structure integrity of the core Mediator or the CDK8 kinase module, suppression of MED13L attenuated their physical interactions and reduced recruitment of acetyltransferase P300 to chromatin via Mediator. Silencing of MED13L therefore diminishes global H3K27ac signals written by P300, activities of enhancer and/or promoters and expression of multiple oncogenes, especially *PRKCA*. Inhibition of *PRKCA* expression potentiates the killing effect of radiotherapy *in vitro* and *in vivo*. Remarkably, high* PRKCA* expression in NSCLC tissues is correlated with poor prognosis of patients received radiotherapy.

**Conclusions:** Our study linking PRKCA to radiosensitivity through a novel mechanism may enable the rational targeting of PRKCA to unlock therapeutic potentials of NSCLC.

## Introduction

Mediator is a large, highly conserved protein complex and functions as a key transcriptional co-activator in eukaryotes 1-4. Human core Mediator has three modules (head, middle and tail) and globally regulates functions of RNA polymerase II (Pol II). As the nexus of Pol II transcription, Mediator could interact with chromatin regulators involved in histone modification, transcription factors (TFs) at enhancers, as well as the Pol II transcription machinery bound at promoters 1, 2, 4, 5. In mammalian cells, long-range chromatin looping interactions between enhancer and promoter sequences seem to be critical for promoting a high-level of gene expression in specific cell types 6-8. Mediator appears to have important roles in stabilizing these chromatin loops via conjunction with chromatin regulators and/or TFs 9, 10. Cancer cells often arise as a consequence of dysregulation of gene expression, such as the upregulated expression of oncogenes. Therefore, Mediator dysfunction is implicated in malignancies 11-13. The cyclin-dependent kinase 8 (CDK8) kinase module reversibly interacts with the core Mediator through MED13, causing further modulation of Mediator's functions 1, 5, 14, 15. For instance, MED13 could interact with MED12 which cooperates with H3K27 acetyltransferases P300 to preserve enhancer activities of multiple genes 5. Although MED13L is the paralog of MED13 16, little is known about its role in cancer cells.

According to the GLOBOCAN estimates of cancer incidence and mortality, lung cancer is most frequently diagnosed cancer (*n* = 2,093,876 new cases, 11.6% of the total cases) and the leading cause of cancer death (*n* = 1,761,007 deaths, 18.4% of the total cancer deaths) in 2018 17. Lung cancer presents a complex disease, which differs by histologic types, i.e. non-small-cell lung cancer (NSCLC) (about 80% of all incident cases) and SCLC (about 20% of all cases) 17. More than 30% of NSCLC patients are diagnosed at locally advanced stage (stage III) 18. Radiotherapy is one indispensable therapeutics in the definitive management for these patients 19. Nevertheless, the median overall survival (OS) of the NSCLC patients treated with radiotherapy remains poor (16-18 months) 20, 21. RTOG0617 Phase III randomized clinical trial indicated that patients with locally advanced NSCLC who received high-dose radiotherapy failed to have survival benefits compared with those receiving standard-dose radiotherapy 22. These results indicate that it might be practical to identify new druggable targets to improve the radiosensitivity of NSCLC cells, but not to simply boost radiation dose.

MicroRNAs (miRNAs) regulate the vast majority of biological events by suppressing the expression of target genes, and their dysregulation plays a crucial part in cancers. Depending on their target genes, miRNAs have been reported to function as either oncogenes or tumor suppressors in lung cancer 23, 24. The importance of miRNAs in radiosensitivity has begun to emerge 25, 26. For instance, ectopic expression of miR-7 could radiosensitize multiple kinds of malignant cells via attenuating EGFR and AKT expression 27. Tumor-suppressive miR-34a could sensitize NSCLC cells to radiation through inhibiting Notch-1 expression and down-regulating the nuclear factor-κB pathway 28. Additionally, exosomes-transported miR-208a can inhibit radiosensitivity of human lung cancer cells by suppressing P21 expression 29. However, the molecular mechanistic detail of miRNAs regulating NSCLC development and radiosensitivity, remains yet to be fully characterized. In this study, we systematically evaluate the radiation-induced miRNAs in lung cancer cells and identified their target genes which are involved in NSCLC response to radiotherapy and progression.

## Results

### Identification of radiation-induced tumor suppressor miR-4497 in NSCLC

**Figure 1:**
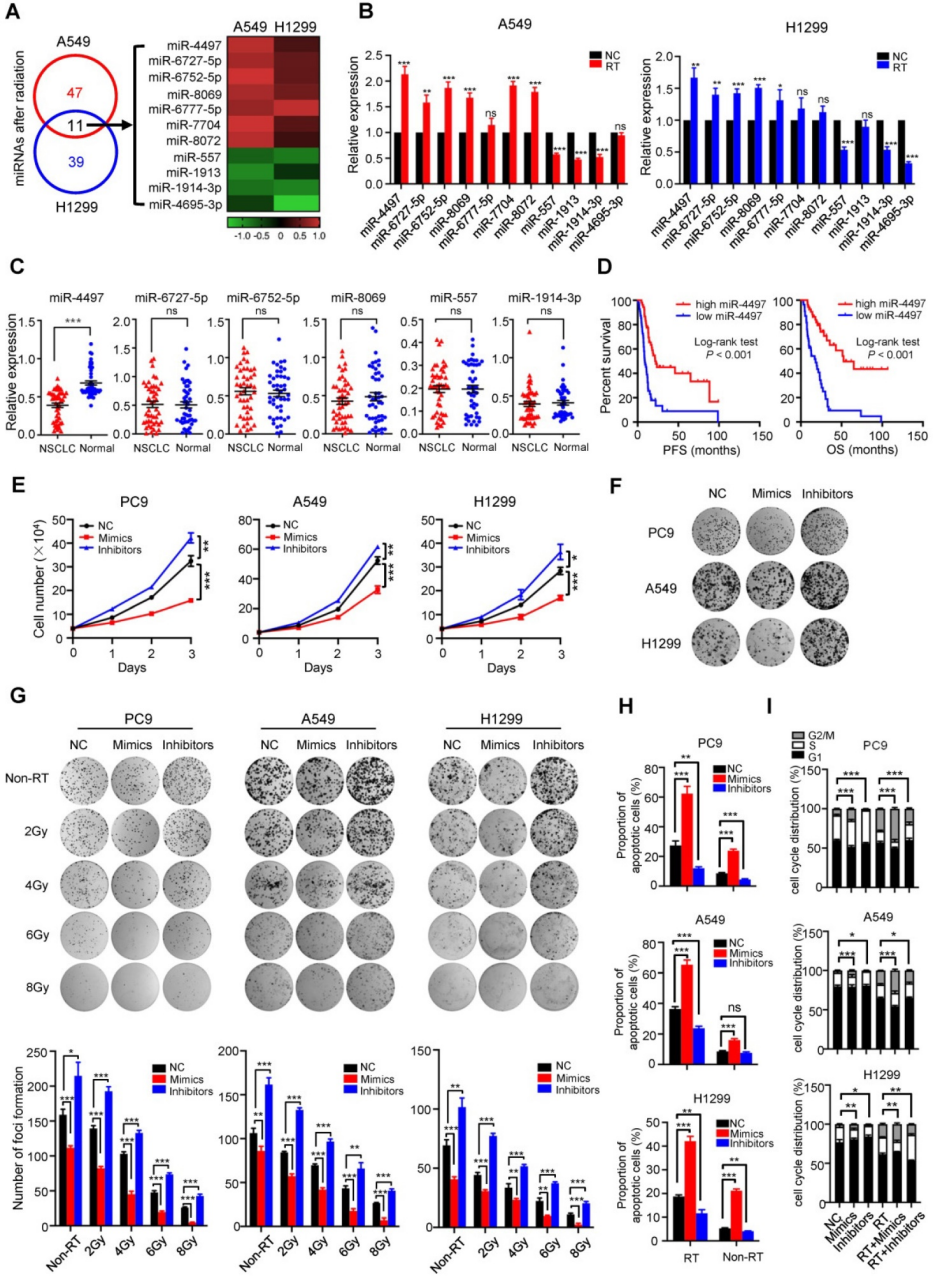
** Radiation-induced miR-4497 functions as a novel tumor suppressor in NSCLC.** (**A**) Human miRNA expression profiling of NSCLC A549 and H1299 cells treated with X-ray radiation (RT) identified eleven significantly dysregulated miRNAs. (**B**) RT-qPCR validation of the eleven candidate miRNAs in A549 and H1299 cells. (**C**) There was markedly decreased miR-4497 expression in NSCLC tissues compared to normal tissues (Shandong cohort) (*n* = 46). (**D**) Low miR-4497 expression was significantly associated with poor progression free survival (PFS) and overall survival (OS) of NSCLC patients treated with concurrent chemoradiotherapy or radiotherapy (Jiangsu cohort) (*n* = 108). (**E**) miR-4497 inhibits proliferation of PC9, A549 and H1299 cells. (**F**) miR-4497 suppresses clonogenicity of PC9, A549 and H1299 cells. (**G**) In colony formation assays, miR-4497 sensitizes NSCLC cells to radiotherapy in a dose dependent manner. (**H, I**) miR-4497 increases apoptosis and G2/M population of NSCLC cells treated with radiotherapy. Data are shown as mean ± SEM. ^*^P < 0.05; ^**^P < 0.01; ^***^P < 0.001 by unpaired Student's *t* test (B, E, G, H, I), by paired Student's *t* test (C), ns: not significant.

To identify radiation-induced miRNAs, we initially profiled human miRNA expression in NSCLC A549 and H1299 cells treated with X-ray radiation (4.906 Gy or 1.529 Gy, median radiation lethal dose) using miRNA microarrays. Indeed, we observed that seven miRNAs were significantly up-regulated and four miRNAs were markedly down-regulated following radiation in both cell lines (*P* < 0.05) (Figure 1A). To confirm the expression changes detected by miRNA profiling analyses, Quantitative reverse transcription PCR (RT-qPCR) were performed to detect the eleven candidate miRNAs. Among the successfully validated miRNAs, we chose four radiation-induced miRNAs (miR-4497, miR-6727-5p, miR-6752-5p and miR-8069) and two radiation-suppressed miRNAs (miR-557 and miR-1914-3p) to be examined in paired NSCLC and normal tissues (*n* = 46, Shandong cohort). As shown in Figure 1C, radiation-induced miR-4497 is the only miRNA with significantly decreased expression in NSCLC tissues comparing to normal tissues (*P* < 0.001). Intriguingly, the elevated miR-4497 expression was evidently associated with longer progression free survival (PFS) and OS of NSCLC patients with locally advanced disease treated with radiotherapy (*n* = 108, Jiangsu cohort) (both Log-rank *P* < 0.001) (Figure 1D).

We further evaluated the role of miR-4497 in NSCLC. Overexpression of miR-4497 could significantly suppress proliferation of PC9, A549 and H1299 cells (all *P* < 0.001). On the contrary, inhibitors of miR-4497 were able to strikingly promote NSCLC cell growth (all *P* < 0.05) (Figure 1E and Supplementary Figure 1A). Similarly, ectopic miR-4497 inhibits colony formation of NSCLC cells (all *P* < 0.001) (Figure 1F). Conversely, miR-4497 inhibitors stimulate clonogenicity of NSCLC cells (all *P* < 0.001) (Figure 1F). We also found that miR-4497 profoundly suppresses the migration and invasion capability of NSCLC cells (Supplementary Figure 1B-1D). These data suggest that miR-4497 acts as a novel tumor suppressor in NSCLC pathogenesis.

We then addressed the possible combined impacts of miR-4497 with X-ray radiation on colony formation, apoptosis and cell cycle control of NSCLC cells. Ectopic miR-4497 sensitizes NSCLC cells to radiotherapy in a dose dependent manner (Figure 1G). In line with this, radiation treated NSCLC cells with inhibition of miR-4497 showed reinforced clonogenicity of NSCLC cells (Figure 1G). Importantly, as radiation does, miR-4497 could significantly induce apoptosis and G2/M cell cycle arrest of NSCLC cells (Figure 1H and 1I). Remarkably, forced expression of miR-4497 strikingly increased apoptosis and G2/M population of NSCLC cells treated with radiotherapy (Figure 1H and 1I; Supplementary Figure 1E and 1F), demonstrating that miR-4497 could enhance NSCLC radiosensitivity.

### MED13L is a direct target gene of miR-4497 in NSCLC

**Figure 2:**
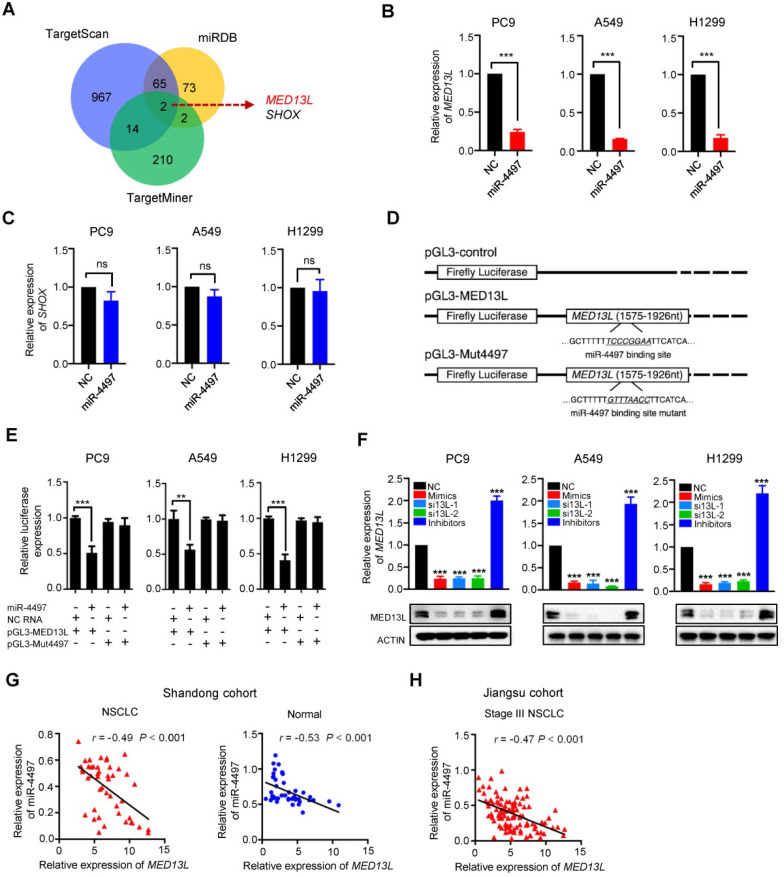
***MED13L* is a direct miR-4497 target gene in NSCLC.** (**A**) Venn diagram of potential candidate target genes of miR-4497 by TargetScan, MiRDB and TargetMiner. (**B, C**) The endogenous expression of *MED13L* but not* SHOX* can be markedly inhibited by miR-4497 in PC9, A549 and H1299 cells. (**D**) Schematic constructions of pGL3-MED13L and pGL3-Mut4497. (**E**) Reporter gene assays indicated that miR-4497 could inhibit luciferase activity of pGL3-MED13L but not pGL3-Mut4497 in NSCLC cells. (**F**) miR-4497 could significantly inhibit *MED13L* protein and mRNA expression in NSCLC cells. (**G, H**) Markedly negative expression correlations between miR-4497 and* MED13L* were observed in NSCLC and normal tissue samples from Shandong cohort (*n* = 46) and Jiangsu cohort (*n* = 108). Data are shown as mean ± SEM. *r* represents the correlation value; ^**^P < 0.01; ^***^P < 0.001 by unpaired Student's *t* test, ns: not significant.

To further investigate the mechanisms of miR-4497 involved in NSCLC, we firstly predicted its potential candidate target genes by integrating results from different algorithms including TargetScan, MiRDB and TargetMiner (Figure 2A). Two overlapped candidate target genes (*MED13L* and* SHOX*) were identified (Figure 2A). Then, we examined the impact of miR-4497 on expression of *MED13L* or* SHOX* in NSCLC cell lines. After transfection with the miR-4497 mimics or negative control RNA (NC RNA) as the negative control, the endogenous expression of *MED13L* but not* SHOX* can be markedly inhibited in PC9, A549 and H1299 cells (Figure 2B and 2C), implying *MED13L* as a potential miR-4497 target gene. Dual luciferase reporter gene assays were conducted to examine the potential direct interaction between miR-4497 and the *MED13L* 3' untranslated region (3'UTR). We firstly subcloned a 352 bp human *MED13L* 3'UTR sequence linked to the firefly luciferase gene and referred the construct as pGL3-MED13L (Figure 2C). Point substitutions were introduced to pGL3-MED13L to disrupt the binding site of miR-4497 in the 3'UTR of the construct and the mutant construct was referred to as pGL3-Mut4497 (Figure 2C). NSCLC cells were co-transfected with pGL3-MED13L and miR-4497 mimics or NC RNA. We found a 49.0%, 43.4% or 59.0% decreased luciferase activity in the miR-4497 transfected group compared to the NC RNA group in PC9, A549 or H1299 cells (all *P* < 0.01) (Figure 2E). However, no significant reduction of luciferase activities caused by miR-4497 was observed in NSCLC cells that were co-transfected with pGL3-Mut4497 and miR-4497 mimics (all *P* > 0.05) (Figure 2E). We then examined *MED13L* mRNA and protein expression in NSCLC cells transfected with the miR-4497 mimics, small interfering RNAs (siRNAs) of *MED13L* (si13L-1 and si13L-2) or NC RNA. Importantly, ectopic miR-4497 could significantly inhibit *MED13L* mRNA and protein expression in all NSCLC cell lines (Figure 2F). In support of the regulatory relationship between miR-4497 and* MED13L*, significantly negative expression correlations between miR-4497 and* MED13L* were observed in NSCLC and normal tissue samples from different patient cohorts (Figure 2G and 2H).

### MED13L promotes malignant phenotypes in NSCLC

**Figure 3:**
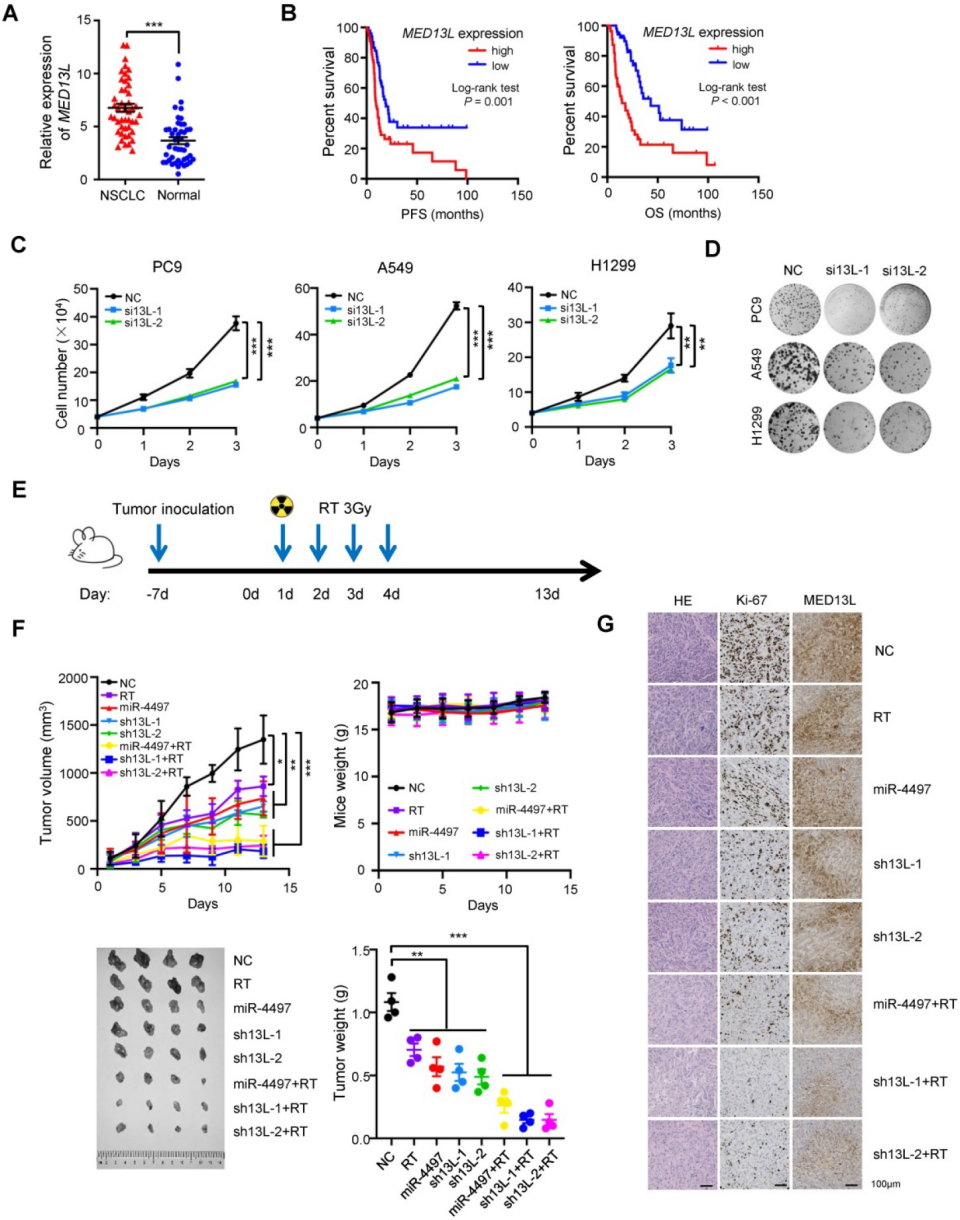
** MED13L promotes malignant phenotypes of NSCLC cells.** (**A**) Significantly elevated *MED13L* expression was observed in NSCLC tissues compared with normal specimens (Shandong cohort) (*n* = 46). (**B**) Concurrent chemoradiotherapy or radiotherapy treated NSCLC patients with low *MED13L* expression exhibited significantly prolonged PFS and OS (Jiangsu cohort) (*n* = 108). (**C**) Silencing of *MED13L* expression (si13L-1 and si13L-2) evidently inhibited PC9, A549 and H1299 cell growth. (**D**) Both si13L-1 and si13L-2 suppressed clonogenicity of NSCLC cells. (**E**) Mice with PC9 xenografts were treated with 4 × 3 Gy X-ray radiation. (**F**) Growth and weights of xenografts with stably ectopic miR-4497 or stably depleted MED13L treated with radiation or not (*n* = 4/group). No differences of mice weight between different groups were observed. (**G**) HE, Ki67 and MED13L staining of tumors from different groups. Data are shown as mean ± SEM. ^*^P < 0.05; ^**^P < 0.01; ^***^P < 0.001 by paired Student's *t* test (A) or by unpaired Student's *t* test (C, F).

MED13L is a subunit of the Mediator complex 16. However, it is still largely unclear how it is involved in NSCLC development. Therefore, we detected *MED13L* mRNA expression in 46 pairs of NSCLC tissues and normal lung tissues (Shandong cohort) 30. There was an evidently elevated *MED13L* expression in NSCLC tissues compared to normal lung samples (*P* < 0.001) (Figure 3A). After detecting *MED13L* expression in biopsy of 108 stage III NSCLC patients treated with concurrent chemoradiotherapy (Jiangsu cohort), we found that NSCLC patients with a relatively high *MED13L* expression exhibited significantly shorter survival time (PFS and OS) compared to cases with low *MED13L* expression (both Logrank *P* ≤ 0.001) (Figure 3B).

We then explored the biological significance of *MED13L* in NSCLC cells. Knockdown of *MED13L* can remarkably suppress viability of NSCLC cells (Figure 3C). In line with the cell viability assays, *MED13L* siRNAs evidently inhibited colony formation of NSCLC cells (all *P* < 0.01) (Figure 3D). To gain insight into the functional relevance of *MED13L* on cell proliferation, we detected how *MED13L* influences apoptosis and cell cycle progression of NSCLC cells. As shown in Supplementary Figure 2A, silencing *MED13L* is able to markedly increase apoptosis of NSCLC cells (all *P* < 0.001). Additionally, *MED13L* siRNAs significantly induces G2/M phase arrest of NSCLC cells (all *P* < 0.01) (Supplementary Figure 2B). We also found that *MED13L* can obviously suppress the migration and invasion capability of NSCLC cells (Supplementary Figure 3C-3F). Together, these data elucidate the oncogenic functions of *MED13L* in NSCLC.

We next evaluated the *in vivo* role of miR-4497 or *MED13L*, alone and in combination with radiotherapy (RT), using NSCLC xenografts. Tumor proliferation of wildtype PC9 xenografts treated with 4 × 3 Gy X-ray radiation (Figure 3E) was evidently inhibited (*P* < 0.05) (Figure 3F). We then generated multiple kinds of PC9 cells with stabilized miR-4497 over-expression or MED13L silencing (shRNAs: sh13L-1 and sh13L-2). Lentivirus-transduced PC9 cells were selected using blasticidin, and either forced-expression of miR-4497 or silencing of MED13L were confirmed by RT-qPCR and/or western blot. Proliferation and colony-forming ability of these cells (miR-4497, sh13L-1 or sh13L-2) were notably retarded *in vitro* (Supplementary Figure 3). Next, we inoculated the miR-4497, sh13L-1 or sh13L-2 PC9 cells into nude mice. Consistent with *in vitro* data, growth of xenografts with stably ectopic miR-4497 or stably depleted MED13L was significantly suppressed compared to the controls (all *P* < 0.01) (Figure 3F). Strikingly, after the tumors stably expressing miR-4497 or stably knocking-down MED13L were treated with radiation, xenografts grew much slower and showed a significant decrease in tumor volume compared to control tumors (all *P* < 0.001). However, there were no differences of mice weight between different treatment groups or the control group. Significantly reduced tumor weights of the miR-4497, sh13L-1 or sh13L-2 stable transfected xenografts were observed compared to controls (all *P* < 0.01). When nude mice bearing miR-4497, sh13L-1 or sh13L-2 stable transfected tumors treated with radiotherapy, xenografts showed a more evidently decreased weights compared to control tumors (all *P* < 0.001). In support of that, the percentage of Ki-67 positive cells was significantly decreased in the tumors of the RT, miR-4497, sh13L-1, and sh13L-2 groups relative to control tumors (Figure 3G). The decreased Ki-67 staining was more manifestly in xenografts of the miR-4497 plus RT, sh13L-1 plus RT, or sh13L-2 plus RT groups compared to control xenografts (Figure 3G). Whereas, there were dramatically decreased MED13L expression in the tumors of different treatment groups relative to control tumors (Figure 3G). Collectively, our results further supported the antitumor activities as well as the radiosensitive effects of miR-4497 or *MED13L* silencing in NSCLC.

### MED13L bridges the CDK8 kinase module with the core Mediator

**Figure 4:**
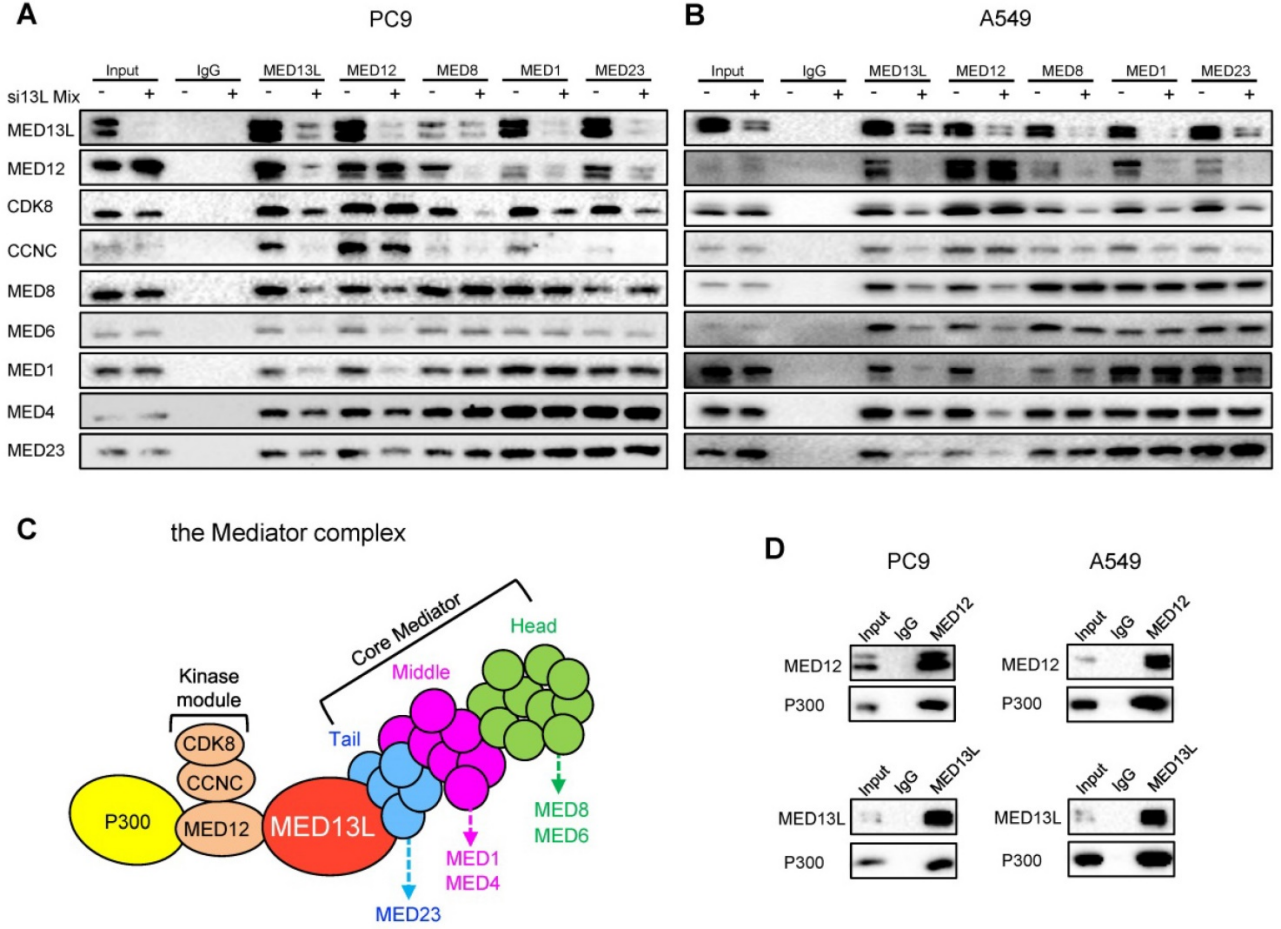
** MED13L bridges the CDK8 kinase module with the core Mediator.** (**A, B**) Immunoprecipitation (IP) experiments using antibodies to MED13L, MED6, MED8, MED1, MED4, MED23, MED12, CCNC and CDK8 in PC9 and A549 cells. (**C**) A carton model indicating the role of MED13L as a vital linker between the core Mediator and the kinase module. (**D**) IP experiments between P300, MED13L and MED12 in NSCLC cells.

To characterize molecular mechanisms underlying MED13L-mediated oncogenic effects, we examined how MED13L impacts the structure integrity of Mediator. Firstly, we performed Immunoprecipitation (IP) experiments using antibodies of MED13L, representative members of the core Mediator (the head module: MED6 and MED8; the middle module: MED1 and MED4; the tail module: MED23) and three components of the CDK8 kinase module (MED12, CCNC and CDK8) in NSCLC cells. Of note, the interaction between the core Mediator and the CDK8 kinase module was significantly attenuated in NSCLC cells after silencing of MED13L (Figure 4A and 4B). For instance, when MED8 (head), MED1 (middle) and MED23 (tail) proteins were immunoprecipitated, the amount of co-immunoprecipitated MED12, CCNC or CDK8 protein (the kinase module) was considerably reduced or almost absent in NSCLC cells after silencing of MED13L. Similar results were obtained for the interaction between MED12 (kinase) and MED8 (head), MED6 (head), MED1 (middle), MED4 (middle) or MED23 (tail) in NSCLC cells with repressed MED13L expression, indicating that MED13L is a vital linker protein between the core Mediator and the CDK8 kinase module (Figure 4C).

After silencing of MED13L in PC9 cells, we found no change for interactions between MED6, MED8, MED1, MED4 and MED23 (Figure 4A), elucidating that interactions among the head, middle, and tail modules remained unaltered. Importantly, no evident change for interactions between MED12, CCNC and CDK8 was observed (Figure 4A), suggesting that suppression of MED13L did not disrupt structure of the kinase module. Consistently, similar results were found in A549 cell after silencing of MED13L (Figure 4B), indicating that MED13L loss does not interrupt structure integrity of the core Mediator or the kinase module.

Although MED12 can physically interact with chromatin regulator P300 in human stem cells 5, little is known about interactions between P300, MED12 and MED13L in NSCLC cells. To investigate if MED13L could recruit P300 via MED12 to Mediator, we performed IP experiments in NSCLC cells (Figure 4D). In line with the previous notion in stem cells, P300 is able to be specifically immunoprecipitated by MED12 or MED13L in both NSCLC cells. Given the physical association between P300, MED12 and MED13L, we hypothesized that recruitment of the MED13L-containing Mediator complex and acetyltransferase P300 might be coupled in H3K27ac modification and regulation of downstream gene transcription.

### Silencing MED13L globally diminishes H3K27ac signals

**Figure 5:**
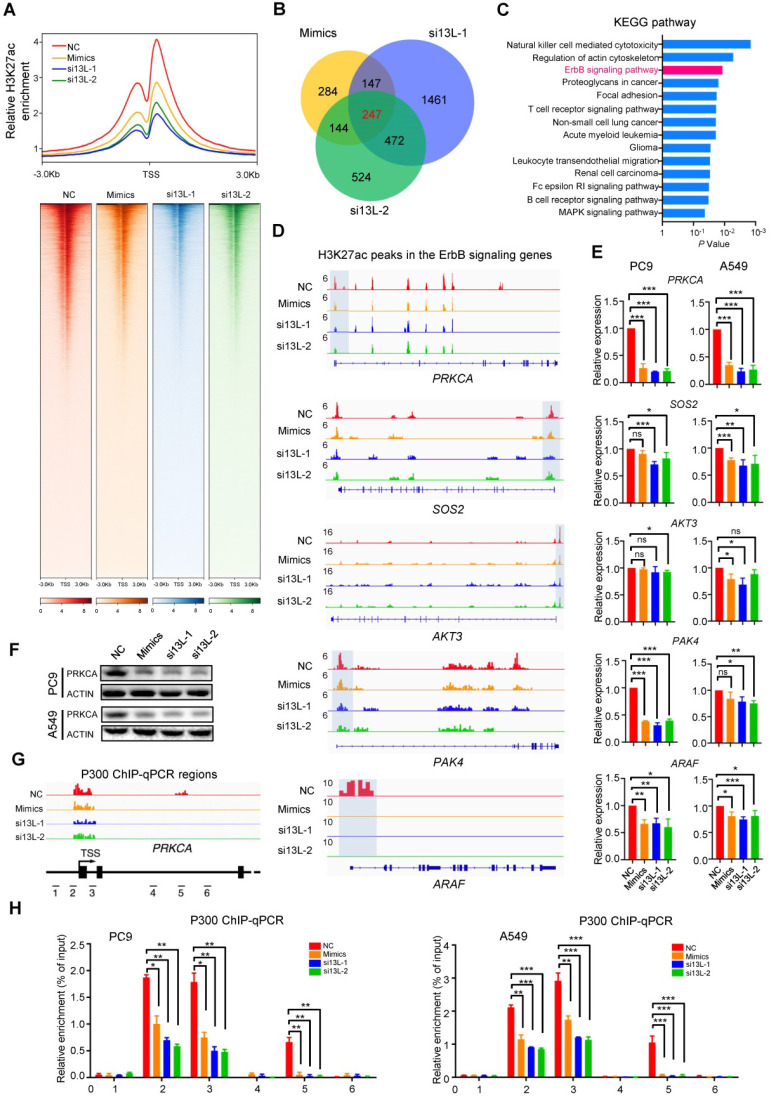
** Silencing MED13L globally diminishes H3K27ac signals.** (**A**) Upper panel: Line plots showing the distribution of H3K27ac ChIP-Seq signals (TSS ± 3 kb). Lower panel: Heatmap showing ChIP-Seq signals at H3K27ac peak regions (TSS ± 3 kb), rank ordered by intensity of H3K27ac peaks based on reads per million mapped reads (RPM). Lines, peaks; color scale of peak intensity is shown at the bottom. (**B**) Integrative analyses of miR-4497 and MED13L (si13L-1 and si13L-2) co-regulated downstream genes with significant changes of H3K27ac modification (*n* = 247). (**C**) The KEGG pathway analyses of 247 genes with evident H3K27ac changes upon MED13L silencing via miR-4497 or its siRNAs. (**D**) ChIP-Seq profiles for H3K27ac at *PRKCA*, *SOS2*, *AKT3*, *PAK4*, or *ARAF* gene. (**E**) RT-qPCR analyses of* PRKCA*, *SOS2*, *AKT3*, *PAK4*, and *ARAF* gene expression in PC9 and A549 cells after forced expression of miR-4497 or silencing of MED13L. (**F**) Ectopic miR-4497 or silencing of MED13L can down-regulate PRKCA expression. ACTIN levels were measured as loading controls in Western blot. (**G**) Chromatin regions 1, 2, 3, 4, 5 and 6 around the TSS of *PRKCA* are detected using P300 ChIP-qPCR. (**H**) ChIP-qPCR results showed P300 can directly bind to chromatin regions 2, 3 and 5, but not regions 1, 4 and 6 in NSCLC cells. Silencing of MED13L by miR-4497 or siRNAs could markedly reduce P300 binding to regions 2, 3 and 5. Data are shown as mean ± SEM. ^*^P < 0.05; ^**^P < 0.01; ^***^P < 0.001 by unpaired Student's *t* test, ns: not significant.

Given the importance of P300 in regulating chromatin structure, we performed histone-modification Chromatin Immunoprecipitation Sequencing (ChIP-seq) of H3K27ac to investigate chromatin changes in NSCLC PC9 cells with ectopic miR-4497 (mimics) or silencing of *MED13L* (si13L-1 and si13L-2). We plotted the normalized tag counts from the H3K27ac ChIP-seq data around the transcriptional start site (TSS ± 3 kb). Intriguingly, when normalized to the same sequencing depth, the H3K27ac signals were much weaker in NSCLC cells with ectopic miR-4497 expression or silenced *MED13L* compared to the control cells (Figure 5A, upper panel). We observed prominently enriched H3K27ac signals around TSS along the genome and lessened H3K27ac enrichment in PC9 cells of the miR-4497, si13L-1 and si13L-2 groups compared to the NC group (Figure 5A, lower panel). Together the results indicate that that silencing of MED13L dramatically impairs mediator-regulated chromatin H3K27ac in NSCLC.

To explore the mechanisms underlying MED13L-mediated downstream signaling, we integrated genes with significant changes of H3K27ac modification in the miR-4497, si13L-1 and si13L-2 groups (Figure 5B). Two hundred forty-seven overlapped candidate genes were identified (Figure 5B). We then performed KEGG pathway analyses of these genes, which revealed that they were enriched for multiple pathways involved in cancer biology, such as the ErbB signaling and NSCLC (Figure 5C). Notably, *PRKCA*, *SOS2*, *AKT3*, *PAK4*, and *ARAF* in the ErbB signaling pathway showed dysregulated H3K27ac around TSS in PC9 cells after ectopic miR-4497 expression or silencing of MED13L (Figure 5D). To evaluate the impacts of MED13L-regulated H3K27ac on expression of these genes, we detected their mRNA levels in NSCLC cells. As shown in Figure 5E, miR-4497 and silencing of MED13L can significantly suppress expression of *PRKCA* and *ARAF* in both NSCLC cell lines. Compared to normal lung tissue samples, mRNA of* PRKCA* but not *ARAF* was significantly upregulated in the NSCLC samples (Supplementary Figure 4A). Hence, we focused on *PRKCA*, also known as protein kinase C alpha, in this study. Ectopic miR-4497 or silencing of MED13L represses PRKCA expression (Figure 5F). Importantly, ChIP-qPCR assays indicate that P300 can directly bind to chromatin regions with H3K27ac ChIP-seq peaks around the *PRKCA* TSS (regions 2, 3 and 5). Silencing of MED13L by miR-4497 or siRNAs markedly reduced P300 binding to regions 2, 3 and 5. However, no obvious P300 binding signals were observed in regions without H3K27ac modification (regions 1, 4 and 6) (Figure 5G and 5H). These data demonstrate that MED13L loss leads to genome-wide attenuated H3K27ac modification around TSS of multiple ErbB signaling genes, especially *PRKCA*.

### Inhibition of PRKCA sensitizes NSCLC to radiotherapy *in vitro* and *in vivo*

Although the role of *PRKCA* in lung cancer has been extensively studied, results are conflicting 31-33. Moreover, it is still largely unknown how *PRKCA* is involved in NSCLC radiotherapy. Firstly, we found that NSCLC cells with stably silencing of *PRKCA* (shP-1 and shP-2) showed markedly impaired cell viability and clonogenic capacity (Supplementary Figure 4B-5D), suggesting a strong oncogenic potential of *PRKCA* in NSCLC.

**Figure 6:**
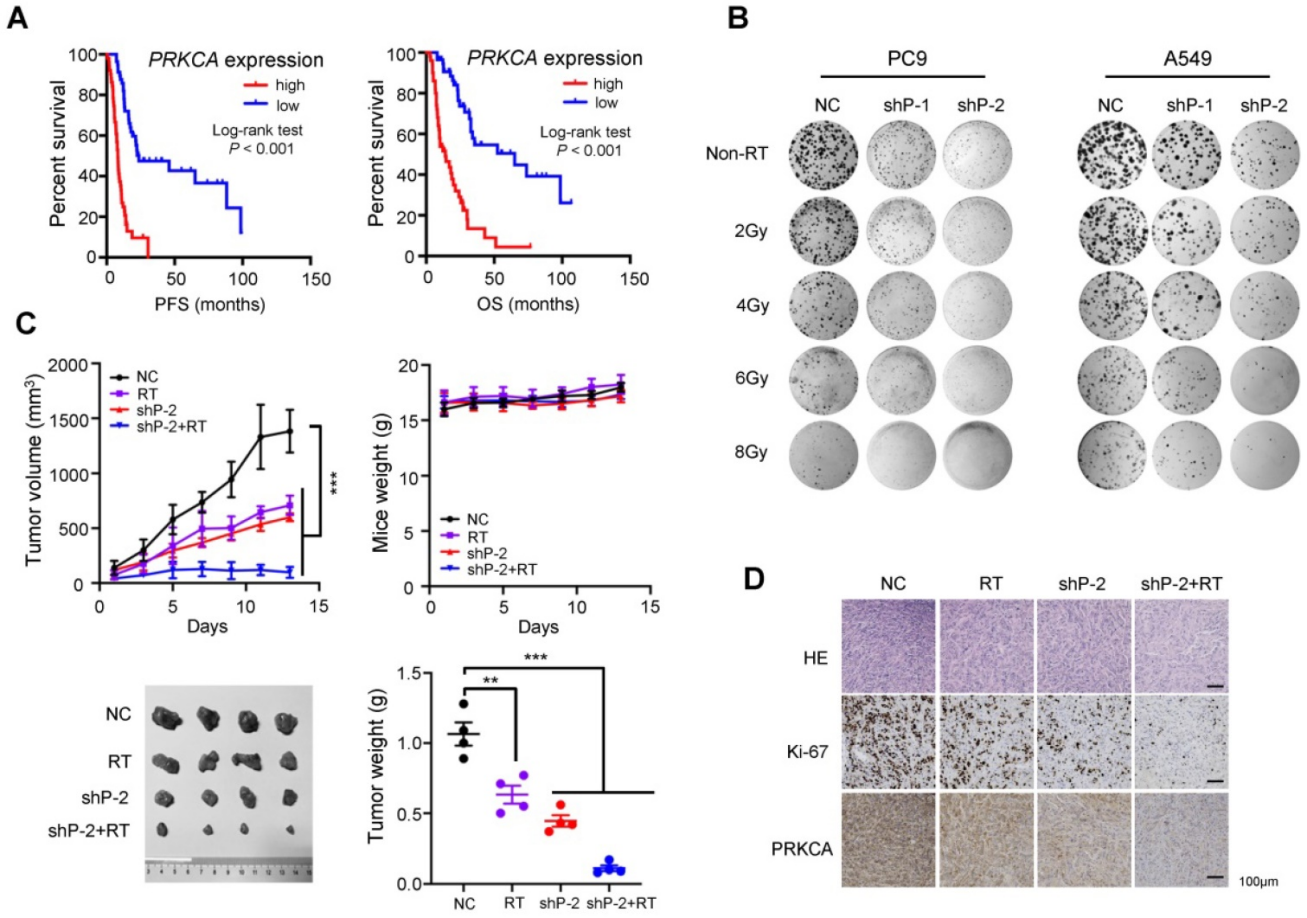
** Inhibition of PRKCA sensitizes NSCLC to radiotherapy* in vitro* and* in vivo*.** (**A**) Radiotherapy-treated NSCLC patients carrying high *PRKCA* expression had a shorter PFS and OS than patients carrying low *PRKCA* expression (*n* = 108). (**B**) Silencing of *PRKCA* sensitized PC9 and A549 cells to radiotherapy in a dose dependent manner via inhibiting their clonogenicity. (**C**) Radiotherapy can remarkably suppress growth of NSCLC xenografts with stably *PRKCA* loss compared to the controls (*n* = 4/group). (**D**) HE, Ki67 and PRKCA staining was performed for tumors from different groups. Data are shown as mean ± SEM. ^**^P < 0.01; ^***^P < 0.001 by unpaired Student's *t* test.

**Figure 7:**
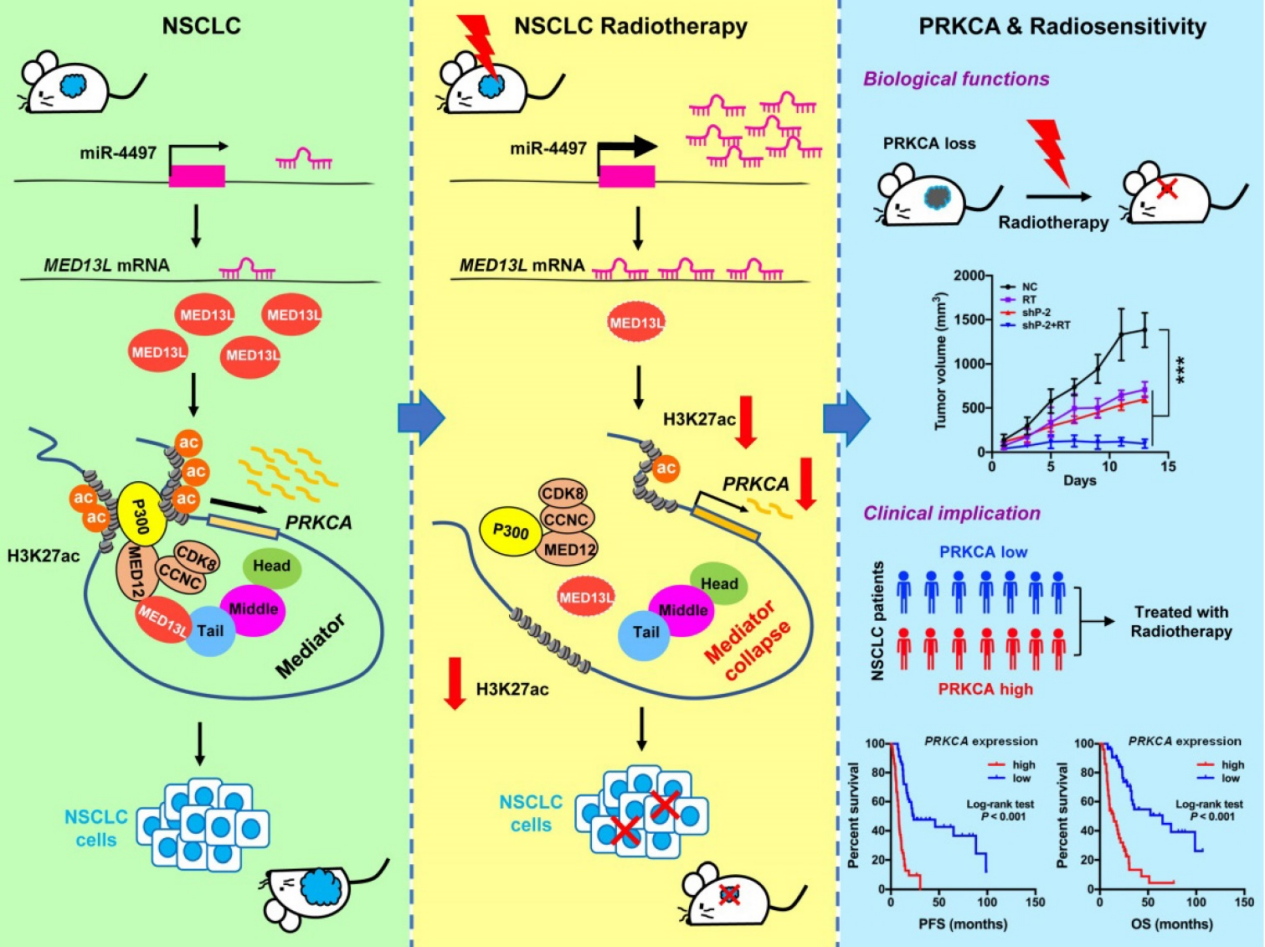
Model for Mediator-regulated epigenetic control of *PRKCA* expression which impacts NSCLC radiosensitivity.

Interestingly, radiotherapy-treated patients carrying high *PRKCA* expression NSCLC had a shorter PFS and OS than patients carrying low *PRKCA* expression tumors (both log-rank *P* < 0.001) (Figure 6A). Importantly, silencing of *PRKCA* sensitizes NSCLC cells to radiotherapy in a dose dependent manner via inhibiting their clonogenicity (Figure 6B and Supplementary Figure 4E). Additionally, the expression levels of MED13L and PRKCA were evidently suppressed in radiation-treated NSCLC cells (Supplementary Figure 4F). In line with this, we found that radiotherapy is able to more remarkably suppress growth of NSCLC xenografts with stably *PRKCA* loss compared to the controls (*P* < 0.001) (Figure 6C). *PRKCA* knockdown also resulted in evident reduction in the percentage of Ki-67 positive cells (Figure 6D). Importantly, tumors with depleted PRKCA treated with radiation showed further decreased Ki67 staining compared to xenografts without radiation (Figure 6D), demonstrating that *PRKCA* might be a promising target to modulate NSCLC radiosensitivity (Figure 7).

## Discussion

More than 70% NSCLC patients with locally advanced or metastatic disease require radiotherapy. To date, efforts to improve NSCLC outcomes with increased radiation dose have not been successful 22. Identification of novel druggable targets that are capable to modulate radiosensitivity of NSCLC cells may provide a way forward. Here we show that tumor suppressor miR-4497 is one of markedly up-regulated miRNAs in response to radiation. As the miR-4497 target gene, MED13L suppression leads to broken link between the core mediator and the CDK8 kinase module which interacts with acetyltransferase P300. Therefore, silencing of MED13L reduced recruitment of P300 to chromatin, diminished global H3K27ac modification, inhibited activities of enhancer and/or promoters as well as decreased expression of multiple oncogenes, especially *PRKCA*. Strikingly, knocking-down of PRKCA could evidently improve NSCLC radiosensitivity (Figure 7).

MED13L was firstly identified as one of the consensus mammalian Mediator subunits using multidimensional protein identification technology 34. In humans, MED13L is similar in size and about 50% identical to MED13 which physically links the CDK8 kinase module to the core Mediator 5, 14, 15, 35, 36, prompting the probability that the less-characterized MED13L may have similar functions. In support of this notion, elevated abundance of MED13 is not adequate to load extra subunits of the kinase module onto the core Mediator 14, suggesting other protein might be involved in controlling assembly of the CDK8 module to Mediator. Importantly, we found that silencing of MED13L disrupts interaction between the kinase module and the core Mediator in NSCLC cells, elucidating the fundamental role for MED13L in maintaining integrity of the Mediator complex.

*MED13L* mutations are associated with intellectual disability and dextro-looped transposition of great arteries 35, 37. Inconsistent results were reported on how MED13L impacts cancer development 36, 38. Glioblastoma T98G cells stably expressing shRNAs of MED13L conferred resistance to the proliferation arrest induced by active Rb expression and showed remarkably stronger clonogenicity compared to the control cells. That is, MED13L may function as a tumor suppressor via promoting Rb/E2F-mediated transcriptional repression and cell cycle arrest 38. On the contrary, in colon cancer, simultaneous depletion of MED13L and MED13 led to decreased cell viability 36 and supported the oncogenic functions of MED13L, which is consistent to our findings in NSCLC.

Histone modifications include acetylation and methylation of various amino acids in histone tails, such as H3K27ac functioning as activating marks. H3K27ac of chromatin is critical for positioning nucleosomes, packaging DNA, controlling the accessibility of chromatin to transcriptional regulators, and modulating gene expression. P300 is one histone lysine acetyltransferase as writer of chromatin H3K27ac. In stem cells, the kinase module (MED12) cooperates with P300 to preserve enhancer activity globally 5. By showing markedly H3K27ac inhibition after silencing of MED13L via ChIP-seq, we have provided evidences that MED13L is likely controlling H3K27ac modification in NSCLC. This notion is further confirmed by the physical interaction between MED13L and P300.

Another interesting finding in the current study is that depletion of PRKCA led to enhanced killing of NSCLC cells by ionizing radiation. PRKCA, a member of the PKC family of phospholipid-dependent serine-threonine kinases, has been implicated in malignant transformation and proliferation. Indeed, human PKCs are encoded by nine genes located in different chromosomes and have been classified into three groups: conventional PKCs (PRKCA, PRKCB and PRKCG), novel PKCs (PRKCH, PRKCE, PRKCQ and PRKCD) and atypical PKCs (PRKCI and PRKCZ) 39, 40. Preclinical and clinical investigations indicated that PRKCA suppression, alone and combination with chemotherapy might be a strategy for NSCLC treatments 31, 32, 41. Several inhibitors of PRKCA, mostly non-specific multitargeted kinase inhibitors, have reached the clinic 42. For instance, a multi-kinase inhibitor midostaurin (PRKCA as one of the targets) has been approved for acute myeloid leukemia therapy. Strikingly, midostaurin could potentially enhance radiosensitivity with a broad therapeutic window 43. Although further investigations should be performed to systematically develop specific inhibitors for PRKCA, the results in this study provide strong evidences that PRKCA is a druggable target for effective radiosensitization of NSCLC.

In summary, we identified a novel model that integrates Mediator-regulated epigenetic control into NSCLC. The fact that radiation can trigger disassemble of the Mediator complex via silencing of MED13L by miR-4497 adds a new layer of gene expression regulation for NSCLC cells to radiotherapy. Results linking PRKCA to NSCLC radiosensitivity through a unique epigenetic mechanism may enable the rational targeting of PRKCA to unlock the therapeutic potential of NSCLC in the clinic.

## Methods

### Cell culture

Human NSCLC A549, H1299 or PC9 cells were cultured in RPMI 1640 medium (Gibco, C11875500BT). Human HEK293T cells were cultured in DMEM medium (Gibco, C11995500BT). All media were supplemented with 10% fetal bovine serum (FBS; Gibco, 1347575). Cells were maintained at 37°C in a 5% CO_2_ incubator and periodically tested mycoplasma negative.

### Genome-wide miRNA expression analyses

Human A549 or H1299 NSCLC cells were treated with X-ray radiation (4.906 Gy or 1.529 Gy, median radiation lethal dose) using an X-RAD 225 small animal irradiator (PXI, US) and harvested after 30 minutes (min) or 1 hour (h). Human miRNA profilling for evaluating the expression of miRNAs in these two NSCLC cell lines before and after radiation was performed using the human miRNA OneArray microarrays (Phalanx, HmiOA7.1) according to the manufacturer's protocol. The array data have been deposited into NCBI's GEO with the accession number GSE147029. The microarray annotations link probe sequences to their targeted mature miRNA sequences in the miRBase database release 21. Raw data was extracted using GenePix 6.0 software (Molecular Devices, US) and was preprocessed and normalized by R statistical software (version 2.12.1) with the limma package and the genefilter package. Siginicantly changed miRNAs were selected for further analyses (*P* < 0.05).

### RT-qPCR

Total RNA was isolated from culture cells or tissue specimens with Trizol reagent (Invitrogen, 94402). To remove genomic DNA, each RNA sample was treated with DNase I (RNase-free) (Thermo Fisher, 18068015). Each RNA sample was then reverse transcribed into cDNAs using PrimeScript^TM^ RT Master Mix (TaKaRa, RR036A). Human miRNAs and U6 were detected with their specific stem-loop RT-PCR primers (Ribobio, Guangzhou, China) using a Quantitative PCR instrument (QuantStudio™ 6, ABI, USA) 30. The relative mRNA expression of *MED13L*, *PRKCA* and other genes were calculated by using the 2^-ΔΔCt^ method. Indicated primers are listed in Supplementary Table 1. Each sample was examined at least in triplicate. PCR product specificity was confirmed by a melting-curve analysis.

### Patients and tissue specimens

There were two cohorts including one hundred fifty-four NSCLC patients recruited in the current study. In Shandong cohort, all patients received curative surgical resection for stage I or II NSCLC in Shandong Cancer Hospital and Institute (*n* = 46, Jinan, Shandong Province, China) between October 2016 and September 2018. Prior to the surgery, no patients received any local or systemic anticancer treatments. Fresh NSCLC specimens and matched adjacent normal lung tissues were obtained from these forty-six patients. The normal tissues were sampled at least 2cm away from the margin of the tumor. The detailed characteristics of all patients have been reported previously 30. In Jiangsu cohort, a total of one hundred and eight biopsy-confirmed stage III NSCLC patients were enrolled in Huaian No. 2 Hospital (Huaian, Jiangsu Province, China) between March 2007 and June 2019. All patients treated with concurrent chemoradiotherapy or radiotherapy alone (Supplementary Table 2). All patients were continuously followed up. PFS was defined as the time from the date of diagnosis to the date of disease progression, or death due to any cause. OS was calculated from the date of diagnosis to the date of death from any cause or the latest follow-up. All subjects were ethnic Han Chinese.

### Transfection of human cell lines

Human miR-4497 mimics, miR-4497 inhibitors and siRNA duplexes for *MED13L* (si13L-1 and si13L-2) were products of Genepharma (Shanghai, China) (Supplementary Table 3). The NC RNA duplex for miRNA mimics, miRNA inhibitors or siRNAs (Genepharma, China) was nonhomologous to any human genome sequence. All small RNAs were transfected with the INTERFERin reagent (Polyplus, 409-10) as reported previously 30. All expression plasmids or reporter gene plasmids were transfected with the jetPRIME reagent (Polyplus, 114-07) or Lipofectamine 2000 (Thermo Fisher, 11668019).

### Lentiviral transduction

Two shRNA hairpins targeting human *MED13L* (sh13L-1 or sh13L-2), *PRKCA* (shP-1 or shP-2) or pre-miR-4497 were were cloned into the pLKO.1 vector. Recombinant lentiviral particles were produced by transient transfection of the plasmids into HEK293T cells. In brief, 4 μg of the shRNA or miRNA plasmid, 3 μg of the psPAX2 plasmid, and 1 μg of the pMD2.G plasmid were transfected using Lipofectamine 2000 into HEK293T cells cultured in 6 cm dishes. Viral supernatant was collected at 48 h and 72 h after transfection and filtered. NSCLC cells were infected with viral supernatant containing 8 μg/mL polybrene, and then selected using 1 μg/mL blasticidin. In these lentiviral transducted cells, the expression levels of MED13L and PRKCA were tested by Western blot and miR-4497 was examined by RT-qPCR.

### Cell proliferation, apoptosis, and cell cycle analyses

A total of 4×10^4^ PC9, A549 or H1299 cells were seeded in 12-well plates. Cells were then transfected with 20 nmol/L miR-4497 mimics, miR-4497 inhibitors, si13L-1, si13L-2 or NC RNA, respectively. Cells were harvested and counted at 24, 48, and 72 h after transfection. NSCLC cells were treated with 8 Gy X-ray radiation at 24 h after transfection and collected after 48 h. Using a FACSCalibur flow cytometer (FCM; BD Biosciences), apoptosis of NSCLC cells was examined with the Alexa Fluor 488 annexin V/Dead Cell Apoptosis Kit (Invitrogen). For cell cycle analyses, NSCLC cells were dyed with PI (Propidium iodide) and detected through FCM (flow cytometry).

### Colony formation assays

NSCLC PC9, A549 or H1299 (1000 cells per well) were seeded into a 6-well cell culture plate. Cells were transfected with various small RNAs (20 nmol/L miR-4497 mimics, miR-4497 inhibitors, si13L-1, si13L-2 or NC RNA). To examine the radiosensitive effects of miR-4497 or silencing of PRKCA, NSCLC cells transfected with miR-4497 mimics or miR-4497 inhibitors or cells stably transfected with shRNAs of *PRKCA* were treated with various dose of radiation (2 Gy~8 Gy). When colonies were visible after 14 days, cells were washed with cold PBS twice and fixed with the fixation fluid (methanol:acetic acid = 3:1). After cells were dyed with crystal violet, the colony number in each well was counted.

### Wound healing and transwell assays

For wound healing assays, a wound was scratched by a 10 μL pipette tip when the cell layer of PC9, A549 or H1299 reached about 90% confluence. NSCLC cells were continued cultured at 37 °C with 5% CO_2_, and the average extent of wound closure was quantified. In transwell assays, the transwell chambers were coated with 60 μL BD Biosciences Matrigel (1:20 dilution) for 12 h in a cell incubator. NSCLC cells transfected with small RNAs were added to upper transwell chambers (pore 8 mm, Corning). A medium containing 10% FBS (650 μL) was added to the lower wells. After 48 h, NSCLC cells migrated to the lower wells through pores were stained with 0.2% crystal violet solution and counted.

### Target gene prediction for miR-4497

Intersecting the results of different bioinformatics prediction algorithms can increase specificity of target gene prediction with low cost 44, 45. Therefore, we integrated the results of three miRNA target prediction algorithms: TargetScan (http://www.targetscan.org/vert-71/), MiRDB (http://mirdb.org/miRDB/) and TargetMiner (https://www.isical.ac.in/~bioinfo_miu/targetminer20.htm). Overall, *MED13L* and *SHOX* were identified by all programs.

### MED13L reporter gene constructs

The sequence corresponding to the wild-type *MED13L* 3'-UTR (1575-1926nt) was amplified with PC9 cDNA using Pyrobest^TM^ DNA Polymerase (TaKaRa, R005A) (PCR primers shown in Supplementary Table 4). The PCR products with blunt ends were ligated into the appropriately digested pGL3-Control. The resultant plasmid, designated pGL3-MED13L, was sequenced to confirm the orientation and integrity. The *MED13L* reporter gene plasmid with mutant miR-4497 binding site was constructed with QuikChange II XL Site-Directed Mutagenesis Kit (Agilent, 200521). These mutant plasmids were confirmed by DNA sequencing and named as pGL3-Mut4497.

### Dual luciferase reporter assays

pGL3-MED13L or pGL3-Mut4497 reporter construct plus 20 nmol/L small RNAs (miR-4497 mimics or NC RNA) were transfected into PC9, A549 and H1299 cells. pRL-SV40 (1 ng) (Promega) containing renilla reniformis luciferase was co-transfected to standardize transfection efficiency. Luciferase activities were detected at 48h after transfection using a Dual-Luciferase Reporter Assay System (Promega, E1910). For each luciferase construct, three independent transfections were done (each in triplicate). Fold change was calculated by defining the activity of pGL3-MED13L as 1.

### Western Blot

Western blot was performed following the standard protocol as previously reported 30. All protein samples were separated in a 10% SDS-PAGE gel. Antibodies against MED13L (1:5000 dilution; Bethyl, A302-421A), MED12 (1:5000 dilution; Bethyl, A300-774A), CDK8 (1:250 dilution; Santa Cruz, sc-13155), CCNC (1:5000 dilution; Bethyl, A301-989A), MED8 (1:500 dilution; Santa Cruz, sc-365960), MED6 (1:500 dilution; Santa Cruz, sc-390474), MED1 (1:5000 dilution; Bethyl, A300-793), MED4 (1:5000 dilution; Abcam, ab129170), MED23 (1:2500 dilution; Bethyl, A300-425A), P300 (1:500 dilution; Santa Cruz, sc-48343), PRKCA (1:1000 dilution; Proteintech, 21991-1-AP) and ACTIN (1:2000 dilution; Sigma, A3854) were used.

### NSCLC xenograft models

To evaluate the* in vivo* role of miR-4497 or *MED13L*, alone and in combination with radiotherapy, a total of 8×10^7^ PC9 cells with stable transfected miR-4497, sh13L-1, sh13L-2 or shP-2 via lentiviral transduction were inoculated subcutaneously into fossa axillaries of five-week-old female nude BALB/c mice (Vital River Laboratory, Beijing, China) (*n* = 4 per group). There were 8 groups including the negative control group, the radiation group, the miR-4497 group, the sh13L-1 group, the sh13L-2 group, the shP-2 group, the miR-4497 plus radiation group, the sh13L-1 plus radiation group, the sh13L-2 plus radiation group, and the shP-2 plus radiation group. During radiotherapy, mice were given a locoregional applied body dose of 3 Gy × 4 consecutive days using a shielding device in an X-RAD 225 small animal irradiator (PXI, US). Tumor volumes were measured every 2 days after tumor volumes equaled to or were greater than 90 mm^3^. Formalin fixed paraffin embedded xenografts were cut in 5 mm thick on polarized glass and firstly hematoxylin and eosin (HE) stained. Anti-Ki-67 (Santa Cruz, sc-23900), anti-MED13L, and anti-PRKCA antibodies were used for Immunohistochemistry (IHC). All procedures involving mice were approved by the Institutional Review Board of Shandong Cancer Hospital and Institute.

### IP

IP was performed between P300 and different components of the Mediator complex, such as MED13L, MED12, CDK8, CCNC, MED8, MED6, MED1, MED4 and MED23. NSCLC cells were lysed in the lysis buffer containing 20 mmol/L Tris-HCl (pH 8.0), 10 mmol/L NaCl, 1 mmol/L EDTA (pH 8.0), 0.5% NP-40, and cOmplete™ Mini protease inhibitor (Roche, 11836170001). Cell lysates were incubated with the indicated antibodies [MED13L (1:100 dilution; Bethyl, A302-421A), MED12 (1:100 dilution; Bethyl, A300-774A), CDK8 (1:100 dilution; Santa Cruz, sc-13155), CCNC (1:100 dilution; Bethyl, A301-989A), MED8 (1:100 dilution; Santa Cruz, sc-365960), MED6 (1:50 dilution; Santa Cruz, sc-390474), MED1 (1:100 dilution; Bethyl, A300-793), MED4 (1:100 dilution; Abcam, ab129170), MED23 (1:50 dilution; Bethyl, A300-425A), P300 (1:100 dilution; Santa Cruz, sc-48343)] or control IgG overnight at 4 °C and at the next day for 2~3h with Dynabeads® Protein G beads (Invitrogen, 10004D). The beads were washed for three times with the lysis buffer, followed by Western Blot.

### ChIP-seq and ChIP-qPCR

For ChIP assays, 6×10^7^ PC9 cells were cross-linked using 1% formaldehyde for 10 min at room temperature. Reactions were quenched by addition of 250 mmol/L glycine for 5 min. Cells were lysed with the cell lysis/wash buffer (150 mmol/L NaCl, 5 mmol/L EDTA [pH7.5], 50 mmol/L Tris-HCl [pH7.5], 0.5% NP-40) plus protease inhibitor for 10 min on ice. Each sample was solved in the shearing buffer (1% SDS, 10 mmol/L EDTA [pH 8.0], 50 mmol/L Tris-HCl [pH 8.0]) plus protease inhibitor and chromatin fragmentation was performed using a Diagenode BioruptorPlus sonicator (30 s on and 30 s off for 12 cycles) to achieve a DNA shear length of 200-500 bp. Solubilized chromatin was incubated with 5 μg anti-H3K27ac antibody (Abcam, ab4729) or IgG control (Invitrogen, 02-6102) overnight at 4 °C on a rotating wheel. Antibody-chromatin complexes were subsequently pulled-down by incubating with Dynabeads® Protein G beads at 4 °C for 4 h on a rotating wheel. Immune complexes were then washed for six times with the cell lysis/wash buffer at 4 °C and then washed twice with the cold TE buffer (Invitrogen, 12090015). Elution and reverse-crosslinking were performed in the elution buffer (100mmol/L NaHCO_3_ and 1%SDS) on a shaker at room temperature for 15 min. After repeating the elution with the elution buffer, antibody-bound chromatin complexes were reversed crosslinked at 65 °C with 5 mol/L NaCl overnight. After reversal of crosslink, each sample was treated with 50 ng/μL RNase A at 37 °C for 30 min and, then, 10 mmol/L Proteinase K at 45 °C for 1h. Immunoprecipitated DNA was extracted with the Min-Elute PCR purification kit (Qiagen, 28004), followed by DNA library preparation and sequencing on the BGISEQ-500 platform. The ChIP-seq data have been deposited into NCBI's GEO with the accession number GSE147120. For ChIP-qPCR assays, the fold enrichment of purified ChIP DNA relative to input DNA at a given genomic site around TSS of *PRKCA* was determined using TB Green® Premix Ex Taq™ I (Tli RNaseH Plus) (TaKaRa, RR820A). ChIP-qPCR reactions were conducted in triplicate and quantified as previously described [46). The six pairs of ChIP-qPCR primers shown in Supplementary Table 5.

### Statistics

Data are expressed as mean ± SEM. The difference between two groups was calculated using the 2-tailed unpaired Student's *t* test or paired Student's *t* test as indicated. The significance of association between miR-4497 and *MED13L* expression was calculated using Spearman's correlation. Impacts of miR-4497 and *MED13L* expression on NSCLC patients' survival was tested by Kaplan-Meier plots and survival durations were analyzed using the log-rank test. A *P* value of less than 0.05 was used as the criterion of statistical significance. All analyses were performed with GraphPad Prism (Version 8.0, GraphPad Software, Inc.).

## Supplementary Material

Supplementary figures and tables.
