# Supplementary material for: MED13L integrates Mediator-regulated epigenetic control into lung cancer radiosensitivity
Source: Theranostics. 2020 Jul 23;10(20):9378–94. doi: 10.7150/thno.48247 (PMC7415817; doi:10.7150/thno.48247)

Supplementary Table 1. qPCR primers used in the study

Genes	Primers	Sequences (5'- 3')
MED13L	Forward	AAATGGGACGCTAACAGG
	Reverse	CCGAACACCACCAACAAT
SHOX	Forward	GAGACCCATTACCCCGACG
	Reverse	ATTCTCTTGTTTGCGGCACTT
PRKCA	Forward	GTCCACAAGAGGTGCCATGAA
	Reverse	AAGGTGGGGCTTCCGTAAGT
PAK4	Forward	GGACATCAAGAGCGACTCGAT
	Reverse	CGACCAGCGACTTCCTTCG
ARAF	Forward	CCTGGCGTTCTGTGACTTCTG
	Reverse	CGGTTGGTACTCATGTCAACAC
SOS2	Forward	ATGTAGAGGAGCGAGTTCAGAA
	Reverse	ATGGTAGTCCACTTTGTACCCT
AKT3	Forward	AATGGACAGAAGCTATCCAGGC
	Reverse	TGATGGGTTGTAGAGGCATCC
ACTIN	Forward	GGCGGCACCACCATGTACCCT
	Reverse	AGGGGCCGGACTCGTCATACT

Supplementary Table 2. Demographic and clinical characteristics of stage III NSCLC patients from Jiangsu cohort

Parameter	Number of patients
Age (years)	70.1 (49.5-89.2)
Gender	
Male	65(60.2%)
Female	43 (39.8%)
Stage	
IIIA	51 (47.2%)
IIIB	57 (52.8%)
Histology	
Adeno	57 (52.8%)
SCC	48 (44.4%)
NSCLC-NOS	3 (2.8%)
KPS Score	80 (60-100)
PFS (months)	
Median	12.5
Event	75 (69.4%)
No event	33 (30.6%)
OS (months)	
Median	19.1
Event	59 (54.6%)
No event	49 (45.4%)

Note: NSCLC: non-small cell lung cancer; Adeno: adenocarcinoma; SCC: squamous cell carcinoma; NSCLC-NOS: NSCLC not otherwise specified; KPS: Karnofsky performance status; PFS: progression-free survival; OS: overall survival.

Supplementary Table 3. RNA oligos used in the study

RNA oligos	Sequences (5'- 3')
miR-4497 mimics	CUCCGGGACGGCUGGGC
miR-4497 inhibitors	GCCCAGCCGUCCCGGAG
MED13L siRNA-1	GCCAAAGACUGCCUCUCAUTT/ AUGAGAGGCAGUCUUUGGCTT
MED13L siRNA-2	GCACAAGCCCAUCUGCAAATT/ UUUGCAGAUGGGCUUGUGCTT

Supplementary Table 4. Primers for *MED13L* 3'-UTR reporter gene constructs

Primers	Sequences (5'- 3')
MED13L-3'-UTR-F	CCGCTCGAGGTACCACCAGTTGAAGGG (XhoI)
MED13L-3'-UTR-R	CTAGAAGCTTATTGGGTTTCTAGGGAAG (HindIII)
Mutagenesis 3'-UTR-F	CTTTGGGGTTGCTTTTTTCCCGGAATTCATCAGAACTTTGA
Mutagenesis 3'-UTR-R	TCAAAGTTCTGATGAATTCCGGGAAAAAAGCAACCCCAAAG

Supplementary Table 5. P300 ChIP-qPCR primers in regions around *PRKCA*

Gene region	Primers	Sequences (5’- 3’)
PRKCA region 1	Forward	ACCTGGAAGCCTTGGAAC
	Reverse	AACTGCCGAAATGCGTAA
PRKCA region2	Forward	GGGCGGCAAGAGCGGAAGAA
	Reverse	GTGCTGGGAACACTGGGCAAGG
PRKCA region 3	Forward	GGCAGGCGGATCACCTAA
	Reverse	CGGAATCTCGCTCTGTCTCAC
PRKCA region 4	Forward	GGGTATAGGGTGAGAAAGT
	Reverse	CTGGAGCATGATGATTGA
PRKCA region 5	Forward	ACTTCACCCACATGACTGT
	Reverse	ATTCACCTCCTGCTTTCT
PRKCA region 6	Forward	GGTGAGATTACTGGGCTAC
	Reverse	GGTGTCTGGGAAGATAAAA

Supplementary Figure 1

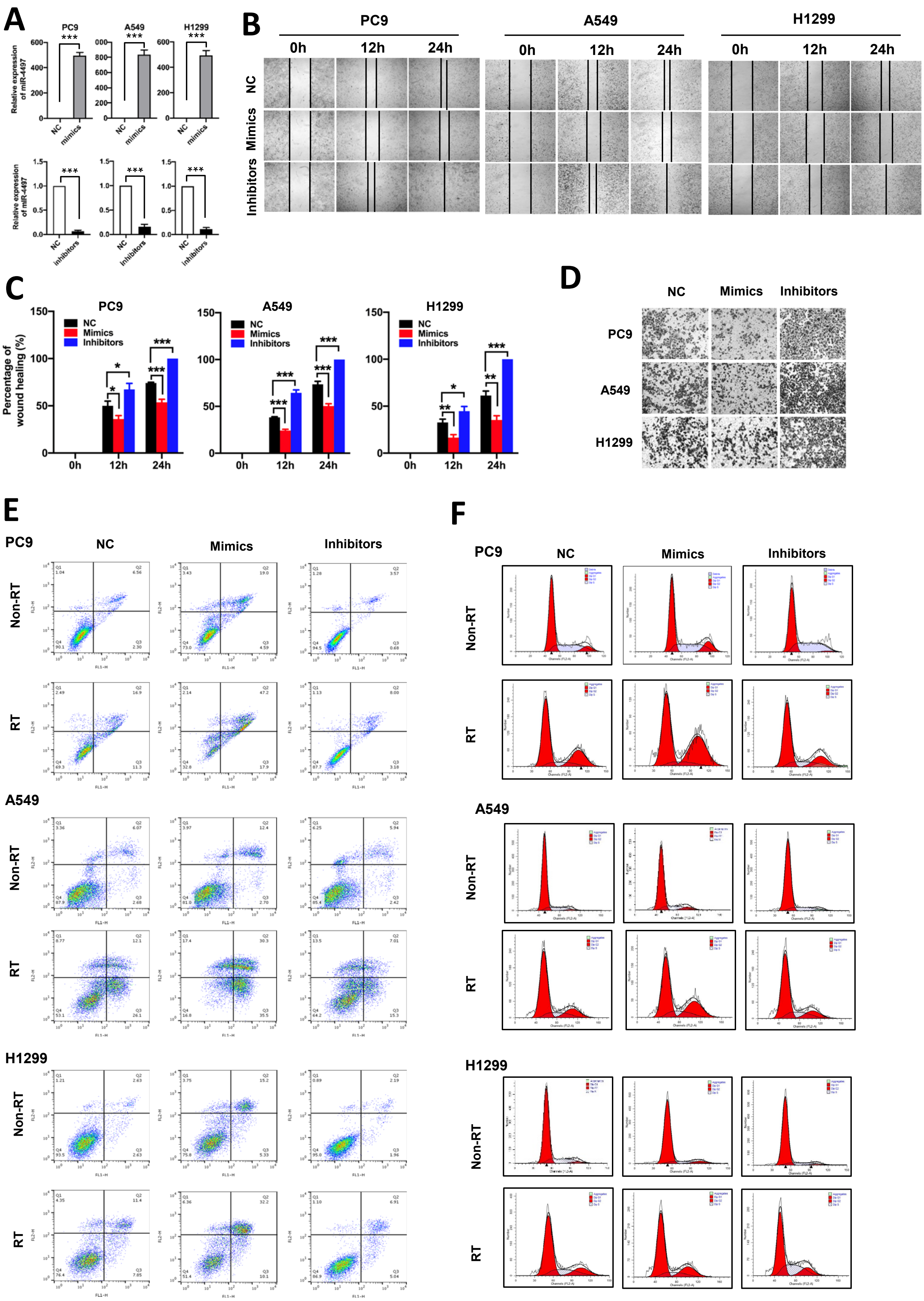

Supplementary Figure 2

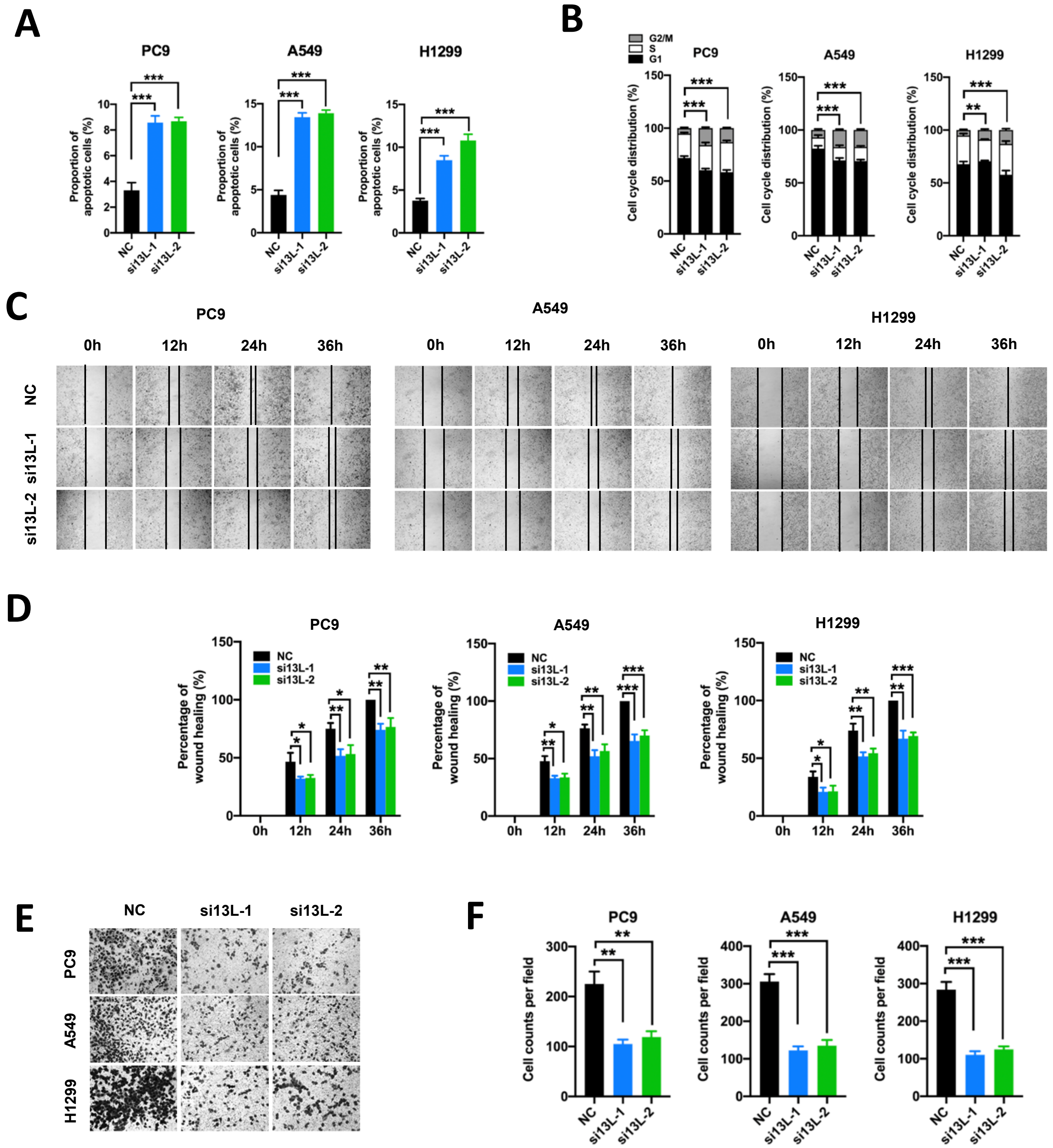

Supplementary Figure 3

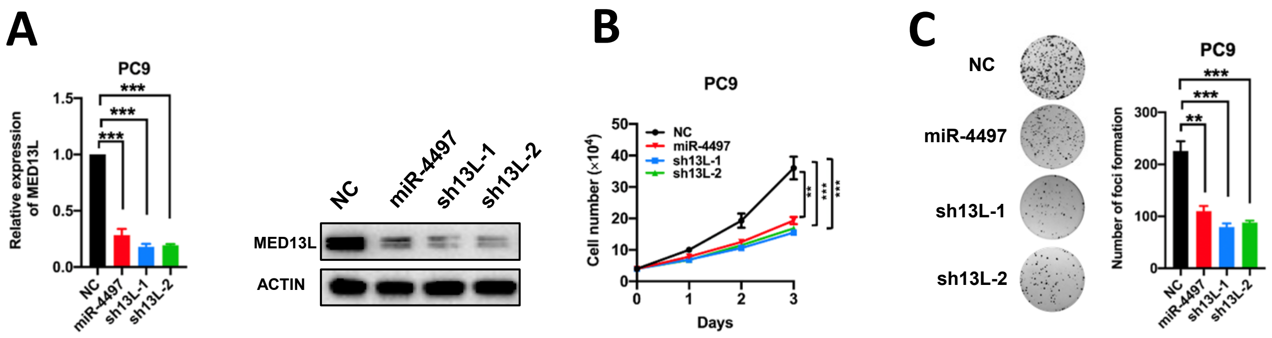

Supplementary Figure 4

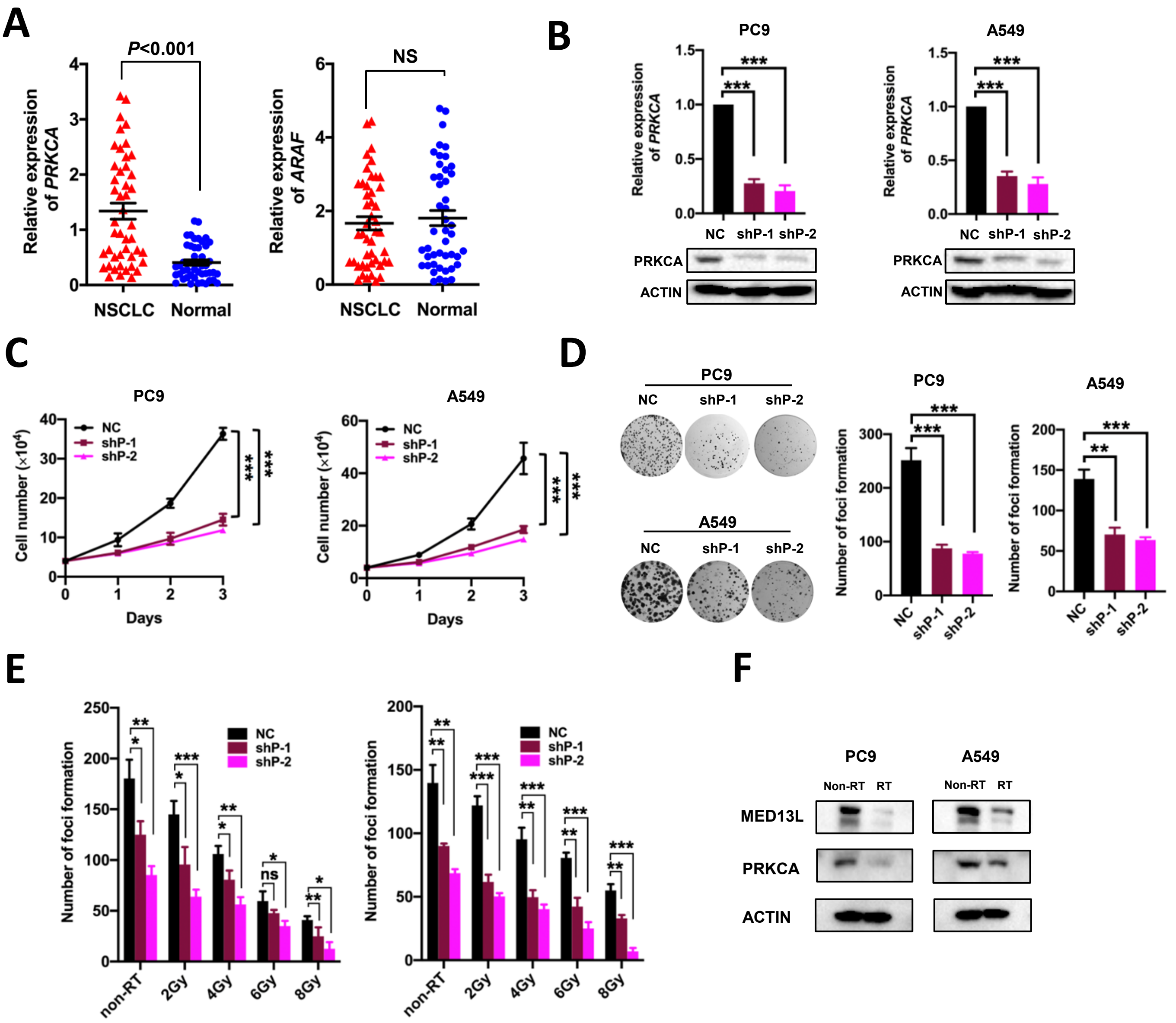

Supplement: Supplementary file 1 — Supplementary figures and tables. [file thnov10p9378s1.pdf]
